# iU-Net: a hybrid structured network with a novel feature fusion approach for medical image segmentation

**DOI:** 10.1186/s13040-023-00320-6

**Published:** 2023-02-21

**Authors:** Yun Jiang, Jinkun Dong, Tongtong Cheng, Yuan Zhang, Xin Lin, Jing Liang

**Affiliations:** College of Computer Science and Engineering, Northwest Normal University, Lanzhou, China

**Keywords:** Medical image segmentation, Deep convolutional neural network, Transformer

## Abstract

In recent years, convolutional neural networks (CNNs) have made great achievements in the field of medical image segmentation, especially full convolutional neural networks based on U-shaped structures and skip connections. However, limited by the inherent limitations of convolution, CNNs-based methods usually exhibit limitations in modeling long-range dependencies and are unable to extract large amounts of global contextual information, which deprives neural networks of the ability to adapt to different visual modalities. In this paper, we propose our own model, which is called iU-Net bacause its structure closely resembles the combination of i and U. iU-Net is a multiple encoder-decoder structure combining Swin Transformer and CNN. We use a hierarchical Swin Transformer structure with shifted windows as the primary encoder and convolution as the secondary encoder to complement the context information extracted by the primary encoder. To sufficiently fuse the feature information extracted from multiple encoders, we design a feature fusion module (W-FFM) based on wave function representation. Besides, a three branch up sampling method(Tri-Upsample) has developed to replace the patch expand in the Swin Transformer, which can effectively avoid the Checkerboard Artifacts caused by the patch expand.

On the skin lesion region segmentation task, the segmentation performance of iU-Net is optimal, with Dice and Iou reaching 90.12% and 83.06%, respectively. To verify the generalization of iU-Net, we used the model trained on ISIC2018 dataset to test on PH2 dataset, and achieved 93.80% Dice and 88.74% IoU. On the lung feild segmentation task, the iU-Net achieved optimal results on IoU and Precision, reaching 98.54% and 94.35% respectively. Extensive experiments demonstrate the segmentation performance and generalization ability of iU-Net.

## Introduction

Benefiting from the rapid development of deep learning, convolutional neural networks (CNNs) have dominated the field of medical image segmentation. Medical image segmentation is a key step in medical computer-aided diagnosis systems. The vast majority of existing medical image segmentation methods are based on the U-shaped network UNet [1], which consists of a symmetric encoder, decoder, and skip connections. The encoder is used as a feature extractor to extract feature information from the image, and the decoder uses the feature information extracted by the encoder to recover the target region in the image. Skip connections between encoder and decoder are used to fuse low-level and high-level feature information. Owing to the clean network structure and excellent performance of UNet, many variants have been derived based on UNet, such as UNet++ [2], Attention_UNet [3], CE-Net [4], CA-Net [5], ResUNet [6] and Double-UNet [7]. The 3D UNet [8] and V-Net [9] with similar structures were proposed for 3D medical image segmentation. Although these networks have been achieved successfully in several medical image segmentation areas, including lung lesion segmentation, dermoscopic image segmentation, and polyp segmentation, CNNs-based approaches typically exhibit limitations in modeling long-distance dependencies due to the limitations of convolutional receptive fields [10], depriving the networks of the ability to adapt to different visual modalities. As a result, network structures based on CNNs typically exhibit weaker performance in the face of target structures which exhibit large inter-patient variation in texture, shape, and size. Pooling layers are often used in CNNs to expand the receptive field. But at the same time, some feature information is lost. In addition, many studies have tried to address this shortcoming by dilated convolution [11], self-attentive mechanisms [12], and pyramid structures [13], but these methods are still inadequate in modeling long-range dependencies.

Up to the proposal of Transformer [14], which was originally used in the field of natural language processing (NLP), Transformer is commonly used for sequence-to-sequence prediction tasks and machine translation. Inspired by the success of Transformer in the field of NLP, researchers have tried to introduce Transformer to the field of computer vision (CV) [15]. Carion et al. [15] proposed an end-to-end transformer structure for object detection, which is the first attempt to introduce the transformer into the CV field. The subsequent proposal of ViT [16] led the peak of Transformer applications in CV. ViT divides images into patches and embeds position encoding, and then makes model pre-training on the large-scale dataset ImageNet, achieving comparable performance to CNN-based methods on image recognition tasks. Liu et al. [17] proposes a hierarchical general framework called Swin Transformer to achieve state-of-the-art performance on image classification, target detection and semantic segmentation tasks. Swin Transformer is built based on Window based MSA (W-MSA) and Shift Window based MSA (SW-MSA). Patch Merging is similar to the pooling layer in CNN, which performs a 2x down-sampling on the feature map to expand the receptive field and increase the number of feature channels, and Patch Expand performs an up-sampling on the feature map to reshape the high resolution image and reduce the number of channels.

To enjoy the benefits of both CNN and Transformer, many studies have tried to combine CNN with Transformer, such as TransUNet [10], TransFuse [18] and MT-UNet [19]. X-Net [20] proposes a hybrid network with dual encoder-decoder for X-shape. Xu et al. [21] propose a multi-dimensional statistical feature network based on the hybrid structure of CNN and Transformer. These networks use a hybrid CNN-Transformer architecture which exploits both the powerful information representation capability of CNN and the encoding ability of Transformer for global contextual information. However, these networks usually have enormous number of parameters and high computational complexity. In the paper, we combine CNN with Swin Transformer, and propose a new network structure, called iU-Net. iU-Net adopts a hybrid architecture of CNN and Swin Transformer, which has the advantages of CNN and Transformer. On the one hand, the addition of Transformer enhances the ability of iU-Net in modeling the contours and boundaries of the lesion region. On the other hand, the local detailed features of the lesion region extracted by CNN compensate for the shortcomings of Transformer in modeling the weak local information, and the two complement each other. Influenced by U-shaped networks [1] and multi-encoder networks [22], iU-Net uses a U-shape network structure with multiple encoders-single decoders. The encoder part includes the primary encoder and secondary encoder, the primary encoder base block is Swin Transformer, the secondary encoder base block is Convolution. The feature information extracted from the primary and secondary encoders, respectively, is fully fused with the features through a wave function-based feature fusion module(W-FFM). The base block of the decoder part is Swin Transformer, and we replace the Patch Expand upsampling method in the decoding stage of Swin Transformer with the proposed Tri-Upsample. We evaluate the performance of iU-Net by 2 typical medical image segmentation tasks, including Skin lesion segmentation on dermoscopic images, Lung segmentation on chest X-rays. Our main contributions are as follows:

1. We propose a multi-encoder U-shape network structure iU-Net with a mixture of CNN and Swin Transformer, including a primary encoder with Swin Transformer as the base building unit and a secondary encoder built with Convolution.
2. We develop a feature fusion module based on wave function representation, which is able to transform feature information from different feature spaces to the same space and then fuse them efficiently.
3. We develop a three branch up-sampling module(Tri-Upsample) to alleviates Checkerboard Artifacts of patch expanding.
4. On the ISIC2018 dataset, the proposed model achieves state-of-the-art performance. At the same time, we do a lot of experiments on the PH2 dataset and lung segmentation dataset to verify the generalization performance of the proposed model.

## Related Works

### CNN-based methods

Most of the traditional medical image segmentation methods are based on boundary detection [23], threshold-based segmentation [24] and machine learning-based algorithms. Although these methods achieved notable segmentation performance, they rely excessively on manual feature selection and the introduction of a priori information [25]. Benefiting from the rapid development of deep learning, CNN-based segmentation methods have dominated the field of medical image segmentation. Especially in 2015, UNet was proposed. A great number of variants have been derived subsequently, such as ResUNet [6], Double-UNet [7]. The 3D UNet [8] and V-Net [9] with similar structures were proposed for 3D medical image segmentation.

### Transformer-based methods

Transformer was first applied to NLP and is usually used for machine translation tasks. Carion et al. [15] first introduced Transformer to the field of cv. In 2021, the Google team proposed the ViT [16] model and achieved comparable performance to CNN in image recognition tasks. Compared with CNN-based methods, the disadvantages of Transformer are the excessive amount of parameters and high computational complexity. However, Swin Transformer [17] solved the problem of excessive amount of parameters by W-MSA and SW-MSA strategies, and achieved hierarchical feature representation by Patch Merging and Patch Expanding. Based on Swin Transformer, many researchers have tried to embed Swin Transformer blocks to U-shaped networks, such as Swin-unet [26], DS-TransUNet [22], and have achieved state-of-the-art performance on several vision tasks, including image classification, target detection, and semantic segmentation.

### CNN-Transformer methods

To enjoy the advantages of CNN and Transformer simultaneously, many works try to combine CNN with Transformer and propose a hybrid network structure of CNN-Transformer, such as TransUNet [10], TransFuse [18], MT-UNet [19], Transformer-Unet [27]. In this work, we try to combine CNN with Swin Transformer and propose a hybrid multi-encoder network structure iU-Net. Unlike TransUNet [10], iU-Net adopts Swin Transformer as one of the base building units of the model, and the computation complexity is well solved.

## Methods

### Architecture overview

**Fig. 1:**
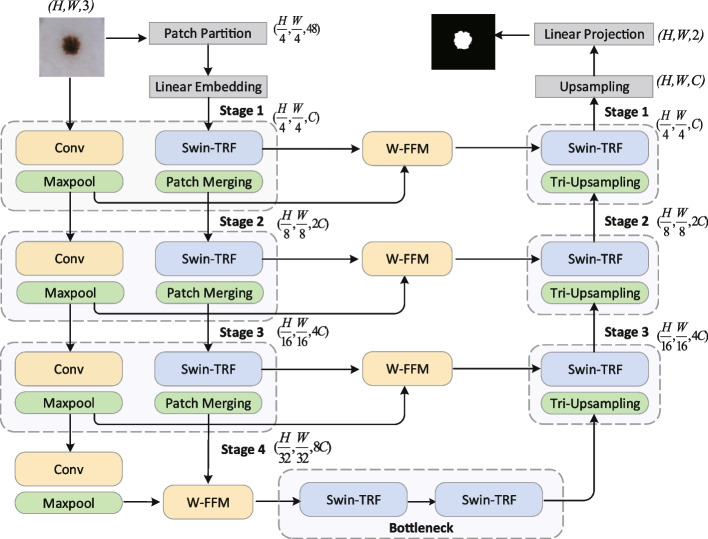
iU-Net network structure

As the network structure is similar to the combination of i and U, the network is called iU-Net. The proposed iU-Net network structure is shown in Fig. 1. The iU-Net consists of 2 encoders, decoder, bottleneck, skip connection and feature fusion module. The encoders are divided into primary and secondary encoders. The base unit of the primary encoder is the Swin Transformer block, and the base unit of the secondary encoder is the Convolution. Firstly, the image passes through the Patch Partition layer, which divides the image into a number of patches. Then maps the number of feature dimensions to an arbitrary dimension (denoted as *C*) through the Linear Embedding layer. Finally patches are input to the primary encoder and go through a series of Swin Transformer blocks with the patch merging. After the patch merging, the feature map is subjected to a 2x down-sampling operation and the number of dimensions of the channels is increased to produce a hierarchical feature map. The secondary encoder uses successive convolution to extract feature information, and a pooling layer is used after each convolution to reduce the number of parameters. The hierarchical features generated by the primary encoder and the features generated by the secondary encoder in the corresponding stage pass through the feature fusion module W-FFM. Then, the fused feature information is input to the decoder part of the corresponding stage through the skip connection to recover the detail information of the image. The decoder consists of successive Swin Transformer blocks and Tri-upsample layers. After the Tri-upsample layer, the pixels of feature information is upsampled by 2x and the number of channels is reduced at the same time. Finally, the feature dimensions are mapped to classes through the linear projection.

### Swin Transformer block

**Fig. 2:**
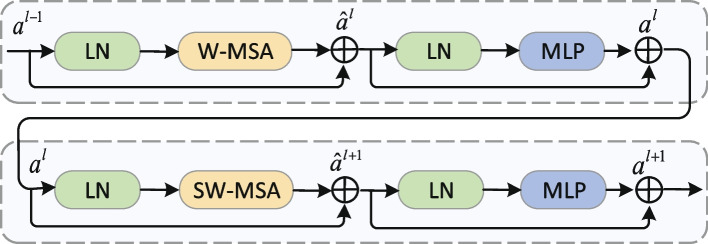
Swin Transformer block

In contrast to the traditional multi-headed self-attention (MSA), which performs self-attention calculation globally, the Swin Transformer introduces Window in MSA, performs local self-attention calculation in Window, and uses the Shifted window technique to enhance the information interaction between windows. Each Swin Transformer block consists of 1 multi-headed attention module, a 2-layer MLP with GELU nonlinearization, 2 LayerNorm (LN) layers and 1 residual connection. The MSA used in the 2 successive Swin Transformer blocks are slightly different: Window-based multi-headed attention module (W-MSA) and shifted window-based multi-headed attention module (SW-MSA). The Fig. 2 shows 2 successive Swin Transformer blocks. The flow of the Swin Transformer block can be expressed as Eqs. (1)-(5).

1 $$\begin{aligned} \hat{z} = W - MSA(LN({z^{l - 1}})) + {z^{l - 1}} \end{aligned}$$

2 $$\begin{aligned} {z^l} = MLP(LN({\hat{z}^l})) + {\hat{z}^l} \end{aligned}$$

3 $$\begin{aligned} {\hat{z}^{l + 1}} = SW - MSA(LN({z^l})) + {z^l} \end{aligned}$$

4 $$\begin{aligned} {z^{l + 1}} = MLP(LN({\hat{z}^{l + 1}})) + {\hat{z}^{l + 1}} \end{aligned}$$

5 $$\begin{aligned} Attention(Q,K,V) = SoftMax(\frac{{Q{K^T}}}{{\sqrt{d} }} + B)V \end{aligned}$$

*Q*, *K*, *V* mean *query*, *key* and *value* metrices respectively. *d* means the dimension of *query*/*key*. *B* represents the embedded position code. $W-MSA$ indicates the window self-attention calculation operation. *MLP* represents the basic block multilayer perceptron of Swin Transformer. $SW-MSA$ indicates shifted window-based multi-headed attention module operation. *Softmax* represents softmax function.

### Encoder

Inspired by [10, 22], we use a dual-encoder U-shape network structure to extract feature information in dermoscopic images. The powerful representation capability of CNN makes it dominant in the field of medical image segmentation, so we choose Convolution operation as the basic unit for building the secondary encoder. The inherent limitations of convolution make CNNs usually exhibit limitations in modeling long-range dependencies. To overcome this shortcoming, we use the Swin Transformer block as the basic unit for building the primary encoder to enhance the ability of modeling long-range dependencies. Given input as $X^{H \times W \times C}$, a sequence of Swin Transformer and patch merging in the primary encoder produces the hierarchical feature map. In the secondary encoder, the input is processed through successive convolution and pooling layers to produce feature information of the same size as the primary encoder. In the same stage, the hierarchical feature representations generated by the primary and secondary encoders are passed through the W-FFM, which fuses features from different spaces. The fused features are input to the decoder section via skip connections.

### Decoder

**Fig. 3:**
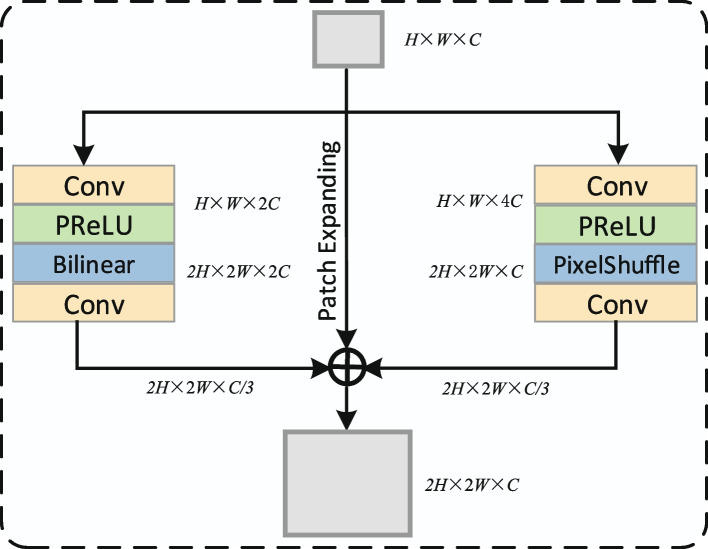
Tri-Upsample module structure

The decoder symmetric with the primary encoder is built based on the Swin Transformer block. The feature information from the bottleneck is processed by several Swin Transformer blocks in turn, while the fused features are input to the Swin Transformer block of the corresponding stage of the decoder through the skip connection. The original SwinUnet recovered images using the patch expanding, which is similar to transpose convolution and is sensitive to Checkerboard Artifacts [28]. Checkerboard Artifacts is the result of deconvolution “Uneven overlap”, which makes one part of the image darker than other parts [29]. To avoid this phenomenon, we use a new upsampling method called Tri-Upsampe. The three branches of Tri-Upsampe use patch expanding, bilinear interpolation and PixelShuffle respectively. The detailed structure of Tri-Upsample is shown in Fig. 3.

### A wave function based Feature Fusion Module

**Fig. 4:**
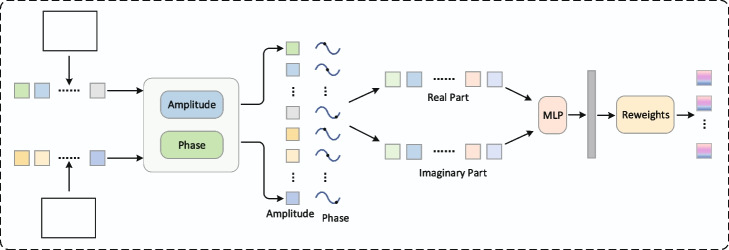
Wave function feature fusion module

The iU-Net is a multi encoder-decoder network model. The decoder consists of a primary encoder and a secondary encoder. The basic block of the primary encoder is Swin Transformer block, and the basic block of the secondary encoder is Convolution. The advantage of multiple encoders is that feature information from different feature spaces can be obtained, but how to aggregate feature information from multiple feature spaces is the core problem of the multi-encoder structure. A direct way is to catenate the 2 different feature maps along the channel dimension and then perform the convolution. But this approach does not capture the global contextual relationship between the different dimensional feature maps and is obviously not the best solution. Given that the features extracted by CNN and Transformer belong to 2 different feature spaces, inspired by [30], we represent the feature information of different feature spaces as wave functions and map them uniformly to the complex domain, and then perform feature aggregation on them in the complex domain, as shown in Fig. 4. At a stage, the features extracted by the primary encoder are denoted as $M_{major}^{m \times H \times W}$, and that extracted by the secondary encoder are denoted as $N_{minor}^{n \times H \times W}$.The spliced features are denoted as $X^{C \times H \times W},C = m + n$, which we divide into non-overlapping tokens and input to W-FFM. Layer Norm is performed first, and then Tokens are represented as waves in terms of amplitude and phase by the dynamic amplitude generation module and phase generation module. As in Eqs. (6)-(8).

6 $$\begin{aligned} |{z_j}| = Amplitude({W^c},{z_j}),j = 1,2,...n \end{aligned}$$

7 $$\begin{aligned} {\theta _j} = \Theta ({W^\theta },{z_j}),j = 1,2,...n \end{aligned}$$

8 $$\begin{aligned} {\tilde{z}_j} = |{z_j}| \cdot {e^{i{\theta _j}}},j = 1,2...n \end{aligned}$$

This is expanded using Euler’s formula, expressed in terms of the real and imaginary parts. The output ${\tilde{o}_j}$ is the complex-value representation of the aggregated feature. After obtaining the aggregated feature information, following the common quantum measurement approach [31], the complex-valued representation of the quantum state is projected into the real-valued observable measurement, and we obtain the real-valued output ${o_j}$ by summing the real and imaginary parts of ${\tilde{o}_j}$ with the weights [30]. As in Eqs. (9)-(11):

9 $$\begin{aligned} {\tilde{z}_j} = |{z_j}| \cdot cos{\theta _j} + i|{z_j}| \cdot sin{\theta _j} \end{aligned}$$

10 $$\begin{aligned} {\tilde{o}_j} = W - FFM{(\tilde{Z},{W^t})_j},j = 1,2,...n \end{aligned}$$

11 $$\begin{aligned} {o_j} = \sum \limits _{} {W_{jk}^t{z_k} \odot } cos{\theta _k} + W_{jk}^i{z_k} \odot sin{\theta _k},j = 1,2, \cdots ,n \end{aligned}$$

## Results

### Implementation Details

In this paper, all methods are implemented using the PyTorch framework. The training process was done on a Quadro RTX 6000 GPU (24GB). The loss functions are the weighted Dice loss function ${L_{Dice}}$ and the Cross-Entropy loss function ${L_{BCE}}$, as in Eq. (12).

12 $$\begin{aligned} Loss = \alpha {L_{Dice}} + (1 - \alpha ){L_{BCE}} \end{aligned}$$

We train the model using SGD optimizer with momentum 0.9, initial learning rate is 0.01, weight decay is 10e-8, batch size is 12, epochs is 300, $\alpha$ is 0.5. The weight parameters pre-trained on ImageNet are used to initialize the model parameters. The size of the input image is set to $224\times 224$, the patch size is 4, and the window size is set to 7.

### Evaluation Metrics

We quantitatively evaluate the segmentation performance of the iU-Net proposed in the paper using Precision, Recall, Dice coefficient, and IoU, as in Eqs. (13)-(16). Precision and Recall are common statistical measures used to evaluate the performance of a binary classification problem. Dice and IoU are used to evaluate the similarity between segmentation results and ground truth. Through Dice and IoU, we can judge the similarity between the prediction and the Ground Truth. The larger the Dice and IoU values, the closer the prediction is to the Ground Truth. Precision indicates the proportion of true diseased pixels in the predicted diseased pixels. Recall indicates how many real diseased pixels are correctly predicted. *TP* represents the correct segmentation of skin lesion pixels, and *FN* is the wrong segmentation of skin lesion pixels. If the segmentation of non-lesion pixels is correctly classified as non-lesion, it is regarded as *TN*. Otherwise, they are *FP*.

13 $$\begin{aligned} Dice = \frac{{2 \times TP}}{{2 \times TP + FN + FP}} \end{aligned}$$

14 $$\begin{aligned} IoU = \frac{{TP}}{{TP + FP + FN}} \end{aligned}$$

15 $$\begin{aligned} Precision = \frac{{TP}}{{TP + FP}} \end{aligned}$$

16 $$\begin{aligned} Recall = \frac{{TP}}{{TP + FN}} \end{aligned}$$

### Datasets

#### ISIC2018

The ISIC2018 dataset [32, 33] includes 2596 RGB images and the corresponding Ground Truth. We randomly divide the images into 2076 for training and 520 for testing. The data augmentation methods include random cropping (224, 224), random rotation $(- \frac{\pi }{6},\frac{\pi }{6})$, horizontal and vertical flipping.

#### PH2

The PH2 dataset [34] is a small dataset consisting of 200 skin lesion images and the corresponding Ground Truth with a resolution of $768\times 560$, which is commonly used to validate the generalization performance of the model. During the training period, the image is resized to $224\times 224$.

#### Montgomery, JSRT & NIH

The JSRT [35] dataset includes 247 chest x-rays of which 154 images are abnormal pulmonary nodule and 93 images are normal. The Montgomery [36] dataset includes 138 chest x-rays, 80 images of normal patients and 58 patients with manifested tuberculosis. The NIH [37] dataset contains 100 chest X-ray images, which include lung diseases with different degrees of prevalence.

**Fig. 5:**
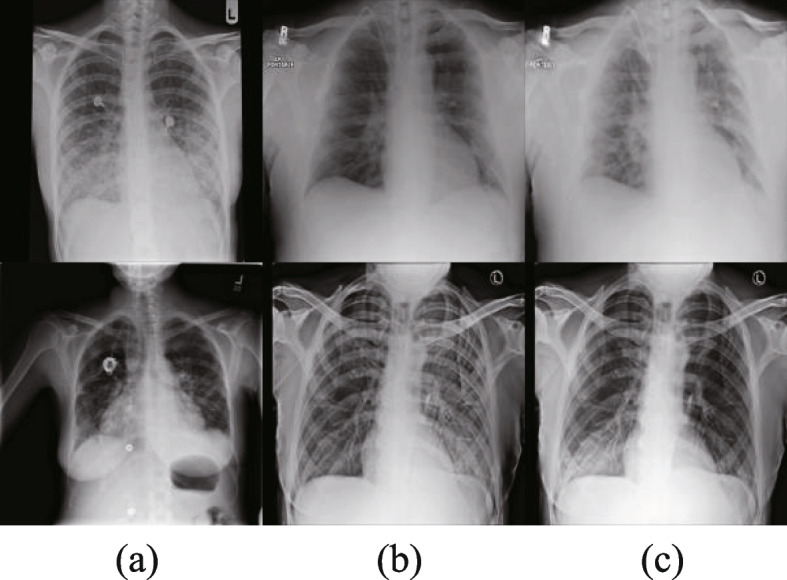
**a**) shows the original image, (**b**) and (**c**) show cases of the data augmentation images

We randomly divide 485 images into 385 for training and 100 for testing. With the data augmentation method proposed in [37], 2400 new images were added, for a total of 2785 training images and 100 test images, as shown in the Fig. 5. During the training period, the image is resized to $224\times 224$.

### Comparison with State-of-the-art Methods

#### Evaluation on Skin Lesion Segmentation

**Fig. 6:**
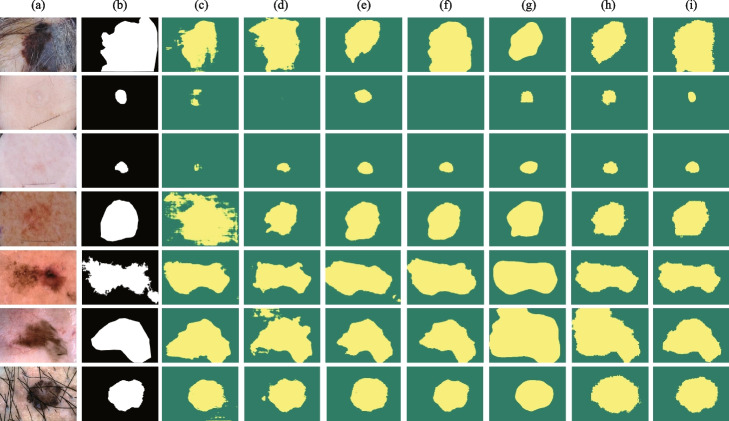
Segmentation results of different models on the ISIC2018 dataset. Column (**a**) original image. (**b**) Ground Truth. (**c**) represents the segmentation result of UNet. (**d**) segmentation result of CANet. (**e**) segmentation result of CENet. (**f**) segmentation result of Atten_UNet. (**g**) segmentation result of TransUNet. (**h**) segmentation result of SwinUnet. (**i**) segmentation result of iU-Net

A comparison of the proposed iU-Net with the state-of-the-art models on the skin lesion ISIC2018 dataset is shown in Table 1. On the ISIC2018 dataset, we reimplemented the models in Table 1 based on the source code, including CNN-based segmentation methods (E.g. UNet, CA-Net), Transformer-based segmentation methods (E.g. TransUNet, SwinUnet) segmentation methods and MLP-based segmentation methods (UNeXt). We implemented TransUNet in the case of using different pre-training models, which include ViT-B_16 and R50-ViT-B_16. Compared with other state-of-the-art models, our proposed model iU-Net achieves the best in 2 evaluation metrics, Dice and IoU, and Precision is second only to SwinUnet and Recall is second only to TransUNet (R50-ViT-B_16). The segmentation results of different models on the skin lesion ISIC2018 dataset are shown in Fig. 6. Based on Fig. 6, the Transformer-based network outperformed the CNNs-based network in segmenting the skin lesion region in close proximity to the healthy skin. Most CNN-based segmentation methods suffer from over-segmentation, which is due to the fact that the information extracted by the convolution operation is local and lacks global contextual information [26].

**Table 1:** Experiment results of skin segmentation for the ISIC2018 dataset

Family	Methods	Year	Dice(%)	IoU(%)	Precision(%)	Recall(%)
CNN	UNet [1]$^{\ast}$	2015	79.89±5.09	71.02±6.69	84.04±4.38	82.01±4.42
	Atten_UNet [3]$^{\ast}$	2018	88.15±8.96	81.21±7.23	85.25±5.85	84.98±5.53
	Channel_Unet [38]	2019	84.82	75.92	94.01	81.04
	ResUNet [6]$^{\ast}$	2019	79.15	70.15	82.43	84.77
	CENet [4]$^{\ast}$	2019	89.53±2.81	82.60±4.53	92.81±4.08	86.76±4.95
	CA-Net [5]$^{\ast}$	2020	90.05±2.43	-	-	-
	PraNet [39]	2021	87.46	80.23	91.28	87.59
	AS-Net [40]	2022	89.55	83.09	-	93.06
	Ms RED [41]$^{\ast}$	2022	87.69±0.53	82.37±0.62	91.87±0.32	88.16±0.58
MLP	UNeXt [42]$^{\ast}$	2022	89.21±0.79	82.1±1.26	-	-
Transformer	SwinUnet [26]$^{\ast}$	2021	88.87	81.67	94.70	86.07
	MedT [43]	2021	87.35±0.18	79.54±0.26	-	-
	ViT-B_16 [16]$^{\ast}$	2021	87.54	80.73	94.20	87.21
	TransUNet(ViT) [10]$^{\ast}$	2021	88.91	81.67	93.05	87.74
	TransUNet(R50) [10]$^{\ast}$	2021	89.71	82.79	94.19	88.21
	FAT-Net [44]	2022	88.9	81.6	-	-
	iU-Net(Ours)	2022	90.12	83.06	94.37	88.07

Model results with “^*^” are reproduced from the published source code. Those with “-” indicate that the corresponding metric results are not provided

#### Cross-validation on PH2

**Fig. 7:**
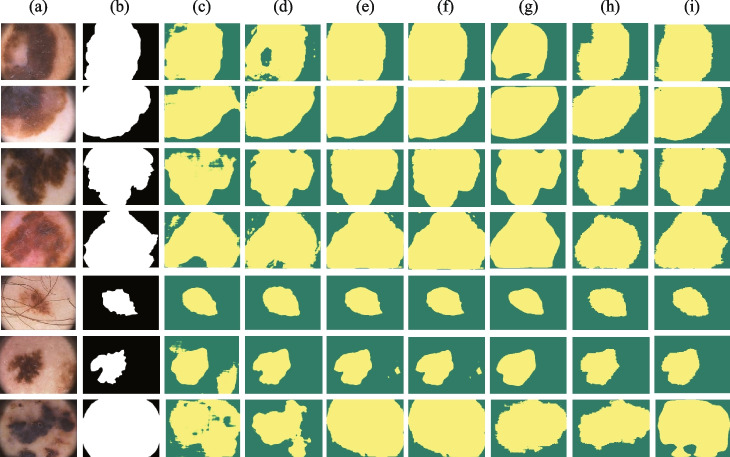
Segmentation results of different models on the PH2 dataset. Column (**a**) original image. (**b**) Ground Truth. (**c**) represents the segmentation result of UNet. (**d**) segmentation result of CANet. (**e**) segmentation result of CENet. (**f**) segmentation result of Atten_UNet. (**g**) segmentation result of TransUNet. (**h**) segmentation result of SwinUnet. (**i**) segmentation result of iU-Net

To further verify the generalization ability of iU-Net to different data distributions, we conducted cross-validation experiments on PH2. The segmentation performance of different models on the PH2 dataset is shown in Table 2. “ISIC2018→PH2” indicates the segmentation performance of the model obtained from the ISIC2018 dataset training on the complete PH2 dataset. The results show that the proposed model iU-Net achieves optimal results in 2 metrics, Dice and IoU, with 93.80% and 88.74%, respectively. Precision is second only to SwinUnet and Recall is second only to TransUNet (ViT-B_16). This indicates the excellent generalization performance of iU-Net. The segmentation results of different models on the PH2 dataset are shown in Fig. 7.

**Table 2:** Experiment results of skin segmentation for the PH2 dataset

Family	Methods	Year	Dice(%)	IoU(%)	Precision(%)	Recall(%)
CNN	UNet [1]$^*$	2015	88.68±7.95	81.85±8.50	83.73±5.94	95.15±5.75
	UNet++ [2]$^*$	2018	91.20	84.35	86.86	96.69
	Atten_UNet [3]$^*$	2018	90.37±8.96	82.21±9.23	85.25±5.85	95.98±5.53
	CENet [4]$^*$	2019	91.75±7.42	85.06±9.74	85.27±5.46	96.70±5.18
	XlSor [37]$^*$	2019	92.95±3.63	87.36±5.66	95.91±2.61	96.58±2.58
	CA-Net [5]$^*$	2020	90.45±8.67	-	-	-
Transformer	SwinUnet [26]$^*$	2021	92.88	87.16	91.58	95.33
	TransUNet(ViT) [10]$^*$	2021	90.85	83.97	86.64	97.14
	TransUNet(R50) [10]$^*$	2021	92.59	86.76	91.31	95.12
	iU-Net(Ours)	2022	93.80	88.74	91.57	96.93

Model results with “*” are reproduced from the published source code

#### Evaluation on Lung Field Segmentation

**Fig. 8:**
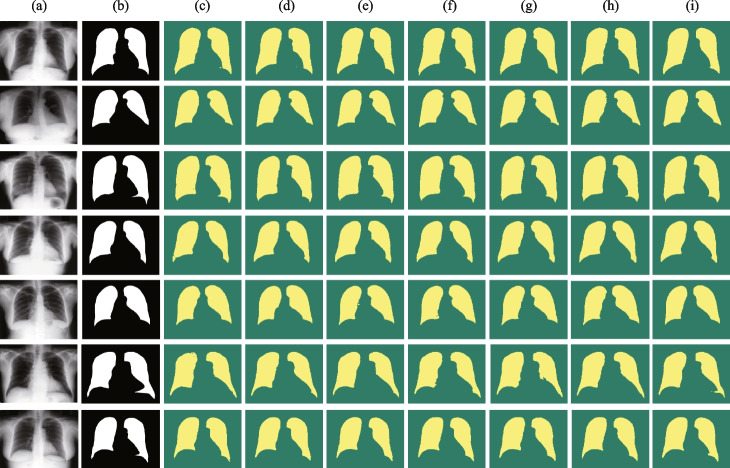
Segmentation results of different models on the PH2 dataset. Column (**a**) original image. (**b**) Ground Truth. (**c**) represents the segmentation result of UNet. (**d**) segmentation result of CANet. (**e**) segmentation result of CENet. (**f**) segmentation result of Atten_UNet. (**g**) segmentation result of TransUNet. (**h**) segmentation result of SwinUnet. (**i**) segmentation result of iU-Net

We evaluate the generalization ability of iU-Net on the lung region segmentation task. The segmentation results of iU-Net with the state-of-the-art models are shown in Table 3. The iU-Net achieved best results in IoU and Precision metrics, which proves the effectiveness of iU-Net for lung image segmentation, with Dice second only to the XLSor [35] model dedicated to lung region segmentation and Recall second only to TransUNet (R50-ViT-B_16). The segmentation results of the different models on the lung dataset are shown in Fig. 8. The segmentation result of iU-Net is closer to Ground Truth than other models. Compared with Baseline (SwinUnet), Dice and IoU are improved by 1.63% and 1.04%, respectively, which indicates that the introduction of the subencoder and W-FFM enables the model to learn more detailed information, and W-FFM can fully integrate detailed information and global contextual information on the same space, which improves the segmentation performance of the model.

**Table 3:** Experiment results of lung feild segmentation

Family	Methods	Year	Dice(%)	IoU(%)	Precision(%)	Recall(%)
CNN	UNet [1]$^*$	2015	95.10±1.33	90.72±2.37	96.38±2.18	90.66±3.18
	UNet++ [2]$^*$	2018	93.48±2.49	88.11±4.10	95.87±1.73	95.00±1.78
	CENet [4]$^*$	2019	96.53±2.81	92.60±4.53	96.76±2.08	94.81±1.95
	Atten_UNet [3]$^*$	2018	95.20±2.36	91.39±2.48	97.42±1.56	91.54±1.07
	XlSor [37]$^*$	2019	97.54	-	97.40	97.73
	CA-Net [5]$^*$	2020	95.95±1.50	-	-	-
Transformer	SwinUnet [26]$^*$	2021	95.58	93.31	96.93	94.34
	TransUNet(ViT) [10]$^*$	2021	96.89	93.98	98.19	95.63
	TransUNet(R50) [10]$^*$	2021	97.03	94.23	98.37	95.02
	iU-Net(Ours)	2022	97.21	94.35	98.54	96.75

Model results with “*” are reproduced from the published source code

### Ablation study

To verify the effect of different factors on the expressiveness of the model, we performed an ablation study based on the skin lesion segmentation task (ISIC2018), including the number of encoders, the upsampling method, and the feature fusion module. Models 1-6 are described as follows.

- Model 1: choose SwinUnet as a baseline.
- Model 2: add sub-encoder based on Model 1.
- Model 3: add Tri-upsampling module based on Model 1.
- Model 4: add sub-encoder and Tri-upsampling module based on Model 1.
- Model 5: add sub-encoder and W-FFM module based on Model 1.
- Model 6: add sub-encoder, Tri-upsampling module and W-FFM module based on Model 1.

**Fig. 9:**
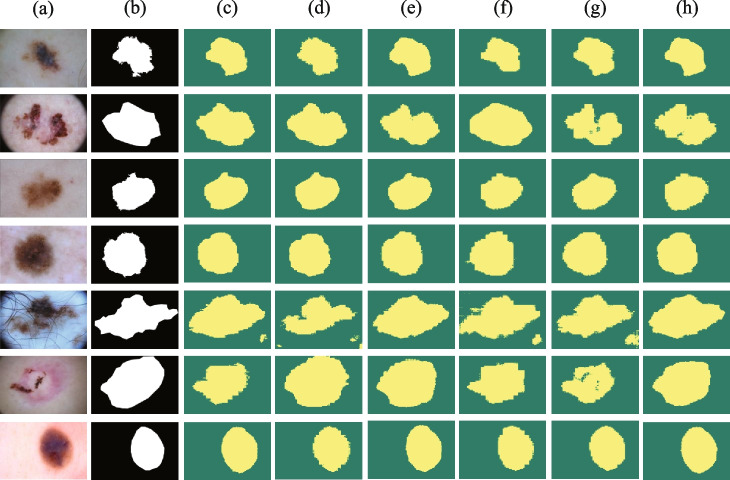
Segmentation results of models 1-6 on the ISIC2018 dataset. (**a**) original image. (**b**) Ground Truth. (**c**) segmentation results of Model 1. (**d**) segmentation result of Model 2. (**e**) segmentation result of Model 3. (**f**) segmentation result of Model 4. (**g**) segmentation result of Model 5. (**h**) segmentation result of Model 6

The experiment results of models 1-6 are shown in Table 4. Compared with model 1, the metrics of Dice and IoU improved by 1.29% and 1.25%, respectively, after adding sub-encoder, which proves that the introduction of sub-encoder has a positive impact on the performance of the model. This is because sub-encoder learns local information that complements the global contextual information extracted by encoder-1. Compared with Model 2, Model 4 uses Tri-upsample module instead of the traditional Patch Expanding, and the IoU is improved by 0.45%. Compared with model 4, the feature fusion method of model 6 is replaced by W-FFM from concatenating to obtain optimal results on Dice, IoU and Precision. The segmentation results of Models 1-6 are shown in Fig. 9.

**Table 4:** Ablation studies of different models on the ISIC2018 dataset. “$\checkmark$” indicates that the corresponding module has been added to the current model and “-” indicates that the corresponding module has not been added to the current model

Model	En-1	En-2	De	T-Up	W-FFM	Dice(%)	IoU(%)	Precision(%)	Recall(%)
1	$\checkmark$	-	$\checkmark$	-	-	87.87	80.67	94.70	86.07
2	$\checkmark$	$\checkmark$	$\checkmark$	-	-	89.16	81.92	94.37	86.78
3	$\checkmark$	-	$\checkmark$	$\checkmark$	-	86.81	80.57	94.42	86.23
4	$\checkmark$	$\checkmark$	$\checkmark$	$\checkmark$	-	89.11	82.37	94.31	87.62
5	$\checkmark$	$\checkmark$	$\checkmark$	-	$\checkmark$	87.24	80.04	94.13	87.19
6	$\checkmark$	$\checkmark$	$\checkmark$	$\checkmark$	$\checkmark$	90.12	83.06	94.52	88.07

**Fig. 10:**
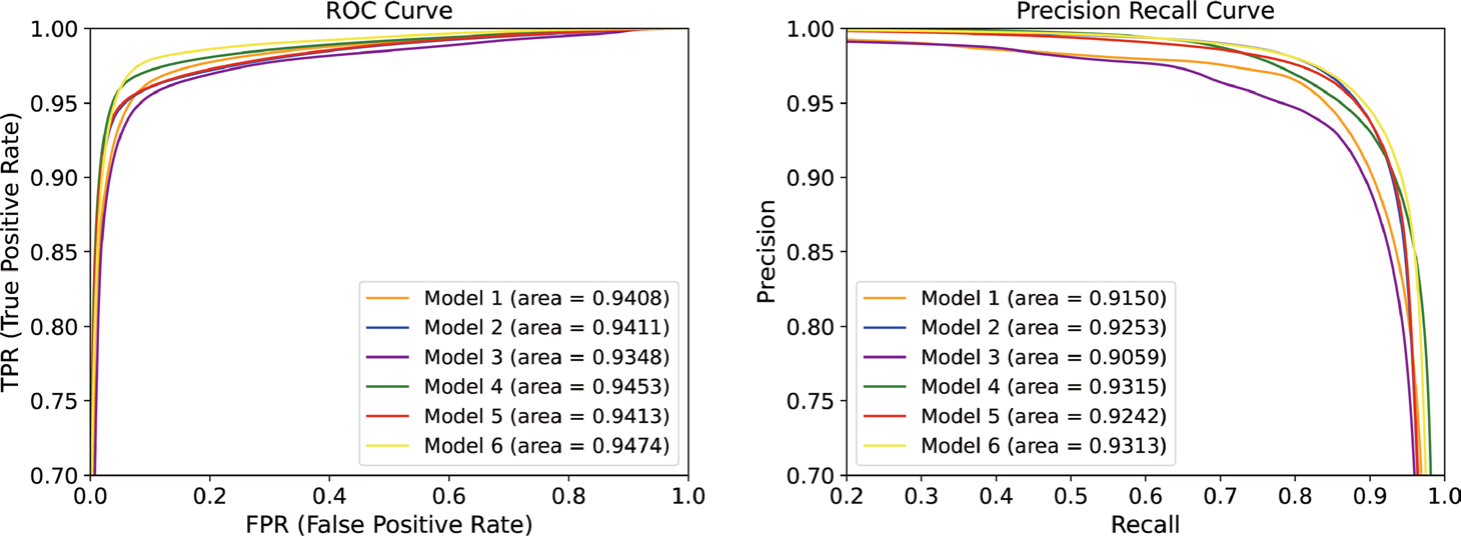
ROC curves and PR curves of the 6 models on the ISIC2018 dataset

To visualize the differences of each model, we plotted the ROC curves and PR curves for models 1-6, respectively, as shown in Fig. 10. It can be seen that model 4 has the highest area of ROC and PR with 94.74% and 94.13%, respectively. The larger area indicates that the segmentation performance of the model is more excellent.

### Visualizations of Decoder Stages

**Fig. 11:**
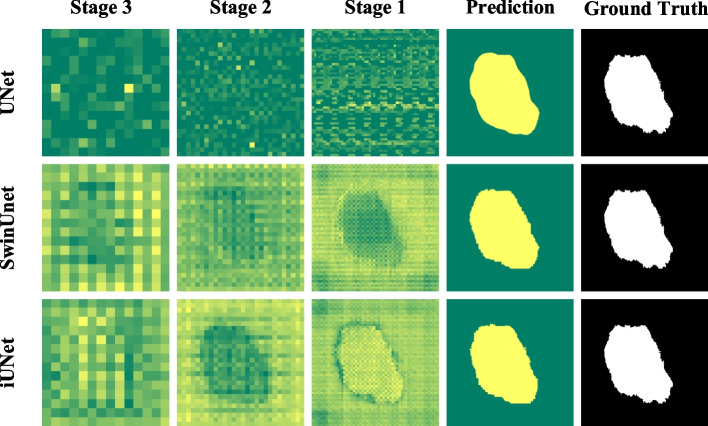
Visualization of different models with different stage features

iU-Net has a stronger ability to capture local information than SwinUnet due to the introduction of sub-encoders. To further verify the semantic recognition capability of iU-Net, we visualized the feature maps for each stage of the decoder part of UNet, SwinUnet and iU-Net, as shown in Fig. 11. Stage represents a stage of the decoder, for instance, Stage3 represents the feature map of the output of the first stage of decoding. Stage1 represents the feature map of the output of the third stage of decoding.

Based on the visualization results, we make the following observations. (1) UNet cannot fully utilize the global contextual information due to the limitation of convolutional kernel, resulting in the features extracted by UNet exhibit localization. Due to the introduction of the Transformer structure, the ability of SwinUnet to model long-range dependencies is significantly improved, so the features extracted by the encoder can provide more global semantic information. (2) Due to the multi-encoder structure of iU-Net, the local information extracted by the sub-encoder can complement the global semantic information extracted by the primary encoder, which makes iU-Net pay more attention to detailed local information when modeling long-range dependencies and makes iU-Net outperform UNet and SwinUnet in segmentation.

## Conclusion

In this work, we combine Swin Transformer with convolutional neural networks to propose a hybrid network with multi-encoder structure for medical image segmentation. In addition, to make full use of the local information features extracted by CNN and the global context information extracted by Transformer, we propose a feature fusion module based on Wave function representation, which can convert feature information from different feature spaces to the same space and fuse them. The iU-Net proposed in the paper is effective for segmentation of dermoscopic images, while the generalizability of iU-Net is verified on the lung feild segmentation task. The combination of Swin Transformer and CNN is effective, and the addition of CNN can improve the performance of Swin Transformer. In future, we will focus on the lightweight of the model. Compared with some models combined by CNN and Transformer, iU-Net is more lightweight, but compared with the parameters of pure convolution neural network and some models based on multilayer perceptron(MLP), iU-Net is not lightweight enough.

## Data Availability

The datasets analyzed during the current study are available from the corresponding author on reasonable request.
